# Re‐evaluating expanding intravenous catheters in medical practice

**DOI:** 10.1002/hsr2.318

**Published:** 2021-07-01

**Authors:** Rigoberto Vazquez, Rishabh Tennankore, Ariella Shikanov, Leonard A. Mermel, Brian Love, Michael L. Burns

**Affiliations:** ^1^ Department of Nuclear Engineering and Radiological Science University of Michigan Ann Arbor Michigan; ^2^ Department of Material Science and Engineering University of Michigan Ann Arbor Michigan; ^3^ Department of Biomedical Engineering University of Michigan Ann Arbor Michigan; ^4^ Department of Medicine, Division of Infectious Diseases Warren Alpert Medical School of Brown University, Rhode Island Hospital Providence Rhode Island; ^5^ Division of Infectious Diseases Rhode Island Hospital Providence Rhode Island; ^6^ Department of Anesthesiology University of Michigan Ann Arbor Michigan

**Keywords:** anaphylactoid reaction, Aquavene, biopolymer, heterogeneous network, intravenous catheter, poly(ethylene glycol), UV cross‐linking

## Abstract

**Background:**

Intravenous catheters are common and essential devices within medical practice. Their placement can be difficult, leading to application of several technologies to improve success. Functionally expanding catheters were once an exciting technology, derailed clinically by hypersensitivity reactions. The exact cause of reactions, attributed to Aquavene catheter materials, remains unknown.

**Aims:**

To reinvestigate functionally expanding intravenous catheters.

**Materials and Methods:**

The history of the functionally expanding intravenous catheter is presented here along with its utility in current medical practice, potential for further investigation, and possible redesign of these once promising devices.

**Results:**

This review demonstrates clinical utility and a lack of definitive cause for failure of the previous functionally expanding intravenous catheter design. As Aquavene materials themselves are commonly considered the cause of hypersensitivity reactions which removed expanding intravenous catheters from the market, this review found several possible substitutes for this material for use in any redesign.

**Discussion and Conclusion:**

The functionally expanding intravenous catheter failed due to hypersensitivity reactions in patients. Alternative materials exist for a possible redesign on this once promising clinical product.

**Abbreviations**
IV: intravenous catheter
PEG: polyethylene glycol
PEG‐DA: polyethylene glycol diacrylate
PEO: polyethylene oxide

## 1 DIFFICULT IV CATHETER PLACEMENT IN MEDICAL PRACTICE

Health professionals understand the difficulties in intravenous catheter (IV) placement. Despite training and expertise, success can be elusive, often requiring multiple attempts by more than one provider, specialized equipment, and/or placement of suboptimum, smaller IVs. Studies have shown 30% to 50% of all IV attempts fail.^1^, ^2^ Common reasons for failure include a patient history of intravenous drug use, dehydration, edema, obesity, patient/provider positioning, and an inappropriate size of IV cannula. Placement failure leads to repeated attempts, and as venous access can cause discomfort and stress to the patient, multiple attempts only cause further patient discomfort and raise both patient and provider anxiety. Difficulty obtaining access also delays the administering of medication, partial or complete loss of the prescribed dose, and more use of materials and provider time, higher costs tied to complications, and lengthier hospital stays.

Methods have been identified and devices created to aid providers in successful IV placement. Methods include improved patient and provider positioning and reducing patient discomfort using lidocaine. Tools to improve successful placement are tied to improving venous imaging, such as infrared vein finders, ultrasound guidance, and transillumination. While helpful in specific situations, these solutions often require additional tools, resources, and training, and their results vary.

Another strategy for improving placement is to use a smaller diameter (larger gauge) IV. Generally, smaller IVs are easier to place when compared to larger IVs as placement may cause less pain, and relative to vessel size, smaller catheters are more favorable for success.^3^ Downsides of using smaller IVs include reduced administration flow rate, higher rates of dislodgement, and increased occlusion or infiltration.^3^ The conflicting need for larger IV access and desire for a high placement success rate led to the creation of *expanding IVs* in the late 1980s. The expanding IV was once a promising product, allowing medical providers the ability to place a smaller sized IV, and when inserted into a vessel, passively expanding to a larger catheter.^4^, ^5^, ^6^ While initially well‐received by the medical community, these products experienced a relatively short commercial run and are no longer available due to safety concerns. In this article, we revisit the development and demise of expanding IVs of the 1980s to 1990s, consider their redevelopment for use in medicine, and we explore materials that could serve as candidates for producing a new irreversible IV polymer expansion system.

## 2 HISTORY AND UTILITY OF EXPANDING IVs

The expanding IV was developed in the Anesthesia Department at Stanford University's School of Medicine in the late 1980s and aimed to aid medical providers in facilitating IV placement and achieving higher catheter flow rates. The history of expanding materials for IV systems involves at least two companies: Becton Dickinson^7^ and Menlo Care Inc.,^8^ though the only marketed “expandable IV catheters” were produced by Menlo Care, an eventual Johnson & Johnson (J&J) subsidiary. To achieve expansion, the Menlo Care catheters all contained a novel polymer blend known as Aquavene, which allowed the diameter and length of the catheter to swell when placed in an aqueous environment such as a vein.^4^, ^5^, ^6^ The change in diameter of the catheter was suggested to be the major factor in the increased inflow, while the increase in length contributed to a slightly decreased flow.^4^ Three products were developed by Menlo Care using this material: the 2 inch in length Streamline peripheral catheter, the 3 to 6 inch Landmark midline catheter, and the 24 inch Centermark catheter. Initially shipped in 1987, these products were removed from the market around 1997 following a series of reported adverse hypersensitivity‐like reactions. The exact cause of these reactions remains unknown.

Case reports questioning the catheter's safety can be found throughout the early 1990s but it was a 1995 prospective report, which found two unexpected adverse reactions among 215 insertions, that seemingly sealed the fate of Aquavene catheters.^9^, ^10^ This report noted that 2 of 251 patients with Landmark catheters inserted had immediate hypersensitivity reactions, however, no such reactions occurred among the 58 580 peripheral Teflon catheters made by other manufacturers inserted by the same nursing team (*P* < .00001, exact 95% lower bound of the odds ratio of 68.9).^9^ From April 1990 through June 1994, a total of 53 adverse reactions were reported to the U.S. Food and Drug Administration that included at least two of the following symptoms: shortness of breath, flushed or mottled skin, back, or abdominal pain.^9^ Twenty‐three of the 53 reactions occurred with flushing the Landmark catheter shortly after IV placement.^9^ Although most of the reactions were transient in nature, 13 of the 53 reactions were severe consisting of cardiac arrest, spontaneous abortion, angioedema of the upper airway, or seizures.^11^ The hypersensitivity‐like reactions were tied specifically to Landmark catheters, but Aquavene itself was never found to be the definitive cause. Biocompatibility studies and catheter residuals did not show evidence of material release or tendencies to invoke immunological or toxicological responses.^12^ There was significant debate over the role of Aquavene in the adverse reactions, with letters finding similar results and calling for additional safety investigations,^13^, ^14^, ^15^ and letters supporting the safe use of Aquavene‐containing products^16^, ^17^, ^18^—however, some investigators may have been funded by MenloCare or J&J. As the adverse reactions often occurred on flushing the catheters and not during placement, there was also speculation that materials being delivered to the patient were the cause, rather than reactions to the catheter materials or their residuals. The exact cause of the reactions was never identified, and marketing and sales suffered from the uncertainty. J&J ultimately recalled all Aquvene‐containing products from the market.

Though the circulation of Aquvene‐containing catheters was short‐lived, the catheters generally received a positive clinical reception.^19^ The concept of an expanding IV was well‐received in the medical community and provider testimony supported their utility: “I've been using Landmark® for years, and I love the things. I've got a four‐year‐old and a one‐year‐old, and if they needed infusion therapy for six weeks, I would insist on a Landmark®.” “The safety—less blood contact—was far superior to other products.” At the time, some providers questioned the findings and hoped for a replacement product: “The studies I read were not conclusive to me. It was a nice system the way they had it set up. If they can use that with a different material [other than Aquavene] that doesn't cause problems, they have themselves onto something.” Much time has passed since expanding IVs were retired, and the field of biomaterials has evolved. It is conceivable the expanding IV could be re‐envisioned with an alternative material – one, which avoids the adverse reactions associated with Aquavene.

## 3 AQUAVENE

**FIGURE 1:**
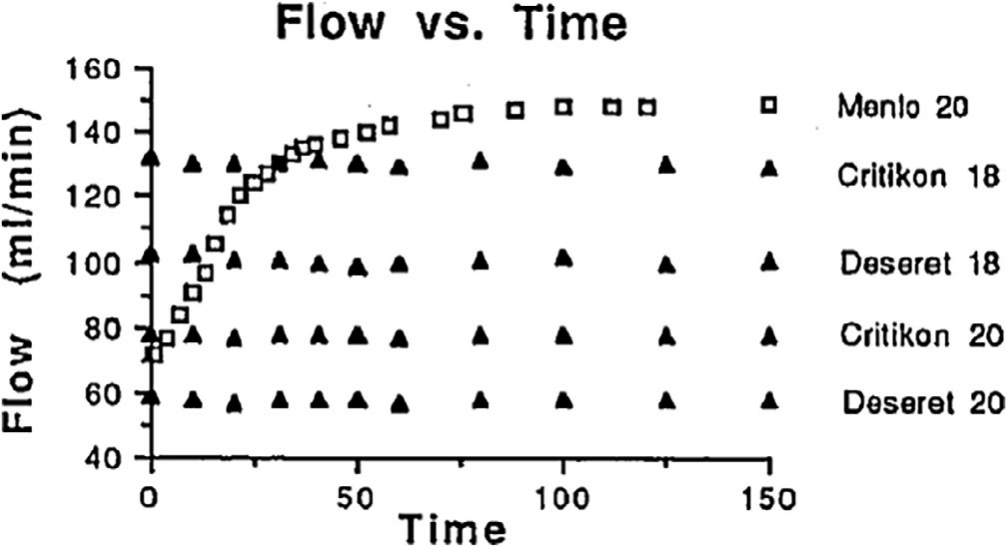
In vitro catheter flow over time comparing Menlo Care 20‐gauge IV catheter (Menlo 20) with four common Teflon catheters. Each point represents the average of three catheters (Fig. 1 from the ASA abstract^5^: Reprinted with permission from the American Society of Anesthesiologists)

Traditional IV catheters are manufactured using materials that are structurally invariant upon insertion. For expanding IV catheters, the key is to make the catheter material sensitive to the insertion environment allowing it to respond by expanding the catheter diameter post‐insertion. This expansion was previously achieved using hydrophilic (water absorbing) resins that swelled when placed in an aqueous environment such as a vein. For Menlo Care, this material was called Aquavene. Early in vitro studies showed that a 20‐gauge IV expanded to an 18‐gauge IV and the flowrate increased 100% over the first hour of simulated insertion (Figure 1).^7^ Upon hydration, the catheters both soften and expand. In vivo results were more modest, with 20‐gauge Streamline catheters achieving a ~26% increase in flow, potentially due to incomplete expansion of segments^4^ or the hydrostatic pressure from the surrounding tissue.

The Landmark midline catheters were composed of an inner layer of polyurethane and an outer layer of the biomaterial Aquavene, a blend of polyurethane and polyethylene oxide (PEO) to which three additional materials are added: antioxidants butylated hydroxyanisole, butylated hydroxytoluene, and the cross‐linker triallyl‐s‐triazine trione. Resins were co‐extruded to form tubing. While there was no evidence of Aquavene materials leaching from catheters upon flushing, it is possible the residuals are rare and simply were not accurately captured in safety studies. It is also conceivable that processing aids like waxes, oils, or other low molecular weight moieties, used to reduce friction in the extruder, could result in other leachable materials being inserted that were not considered part of the formulation.^20^ Residuals and processing aids must be thoroughly investigated in any catheter design and it remains uncertain if leached materials caused the reactions witnessed in Aquavene‐containing products based on reactions occurring during initial flushing of the catheters.

Nonexpanding catheters are commonly made from silicones, polyvinyl‐chloride, or fluorinated polymers like Polytetrafluoroethylene (Teflon). These materials are mechanically strong, flexible, and are sufficiently inert for medical usage. Catheters must also be generally biocompatible otherwise they can cause irritations or T‐cell‐activated allergic reactions, such as phlebitis or anaphylaxis. In addition, novel components such as magnetized stylet wires made up of a neodymium‐iron‐boron mixture used for peripherally inserted central catheter insertion may be associated with anaphylaxis and anaphylactoid reactions.^21^ The tubing must also maintain integrity with myriad medications, blood products, and chemicals that are delivered through a vascular catheter. Compounds such as contrast dyes, opioids, and albumin differ in composition and any of these could lead to protein adsorption or clogging. Activation of the clotting cascade and clot formation downstream from the catheter is another consideration. The broad goal of any catheter is to not activate platelets and T cells, resulting in clots or triggering inflammation in the catheterized vein.^2^ Finally, intravascular devices, especially indwelling catheters, carry nontrivial risks for infection and that risk rises the longer they are inserted. When designing any new form of expanding IV catheter, one must consider all these important elements in addition to identifying compatible hydrophilic resins as tissue responsive IV candidates.

## 4 THE SEARCH FOR ADDITIONAL EXPANDING IV MATERIALS

In considering expanding catheter designs it is desirable to maintain the existing functionality and stability of existing nonexpanding IV catheters, including expanded fluoropolymers, plasticized polyvinyl‐chloride, polyethylene, latex rubber, silicones, and polyurethanes. Most IV catheters are designed to maintain their form. The resins are often so hydrophobic so as to be completely nonresponsive and nonswelling. On the other hand, silicones and polyurethanes can be formulated with hydrophilic components. As such, these compounds were the obvious choices in the original Aquavene catheters. Depending on the fraction of soft, hydrophilic segments in the polymer resin formulation, the swelling feature of these compounds can be tuned up or down. In considering the design space for new IV catheter resins, two strategies seem most appropriate for expanding IV design: (a) add hydrophilicity into a homopolymer, or (b) copolymerizing a more aqueous sensitive resin with other biocompatible resins (the method in which Aquavene was produced).

As the safe use of Aquavene is in doubt, we consider other candidate materials that could have an appropriate level of aqueous sensitivity to achieve clinically meaningful expansion. Considering general biocompatibility as a given requirement, condensation polymers based on polyester, polyamide, and polyurethane moieties and addition polymers with sufficient sidechain hydrogen bonding seem like plausible candidates to consider (Table 1).

**TABLE 1:** Candidate resins with promising properties for intravenous catheter expansion

Synthetic polymers	Degree of swelling (%) in DI water	Features
Polyvinyl alcohol^22^	3‐25	• Low‐cytotoxicity • *Strong* Mechanical properties
Polyethylene glycol (PEG)^23^	280‐870	• Low‐cytotoxicity (only toxic @ >50% concentration) • *Weak* Mechanical properties • *Molecular weight ranges: 200‐3400*
Poly(2‐hydroxyethyl methacrylate)^24^	40‐74	• Low‐cytotoxicity (99% cell viability over 60 days) • *Moderate* Mechanical properties
Polyacrylamide^25^	10‐58	• Low‐cytotoxicity (monomer causes toxic effects) • *Weak* Mechanical properties
Polyurethane^26^	12‐60	• Low‐cytotoxicity • *Moderate* Mechanical properties • PEG‐based soft blocks added to regulate strength, stiffness, and swell ability

Natural polymers	Degree of swelling (%) in DI water	Biocompatibility
Citric acid^27^	402‐620	• *Moderate*‐Cytotoxicity (Dose‐dependent) • *Weak* Mechanical properties • Polyglycerol is commonly added to limit the pore sizes for holding water
Hyaluronic acid^28^	1500‐1725	• Low‐Cytotoxicity (Depends on concentration; 5%‐30%) • *Weak* Mechanical properties • Divinyl sulfone can be added as a cross‐linker to improve swelling properties
Chitosan^29^	590‐1020	• Low‐Cytotoxicity (No side effects) • *Weak* Mechanical properties • Glutaraldehyde (GA), Starch, or Epoxy can be added to form stronger chitosan hydrophilic networks
Alginic acid^30^	75‐212	• Low‐Cytotoxicity (No side effects) • *Weak* Mechanical properties • Cross‐linkers (eg, PEG) are added to compensate for loss of hydrophilic character

*Note*: The degree of swelling is given as a general range that is formulation dependent.

Ideally, these materials would be extruded into tubular forms that would not leak, rupture, or otherwise degrade during use. Catheters may exist for days to weeks in the in‐hospital care setting, but for in‐patient procedures, such as surgery, idealized expansion should occur on the scale of <2 hours. Aquavene catheters achieved significant expansion in 1 hour (Figure 1) and fully expanded in just over 2 hours.^7^ The inner diameter expansion must also be significant enough to increase fluid throughput. The inner diameter of an 18‐gauge catheter is ~30% greater than a 20‐gauge. Given that in vivo responses were smaller than in vitro, a lower threshold of ~50% expansion seems justified to consider for successful design. Too much expansion could also pose a problem potentially causing infiltration, trauma to the vessel wall, or the tube could become so flexible and soft that the catheter could bend, distend, rupture, or kink near the insertion site. Highly swollen resins might also increase the migration potential of small molecules sequestered within them, creating a similar condition to what was perceived a problem for Aquavene. Thus, there exists a design compromise between the scale of the swelling response and mechanical stiffness.

To redesign the expanding IV catheter, one could begin with the candidate materials in Table 1, starting with a catheter as a single resin solution and refining the formulation as warranted. Focusing first on free radical polymerized polymers and how much the side chains can be hydrolyzed to achieve desired swelling response. These materials can have polymer backbone elements that are more sensitive to aqueous swelling responses, as most all‐carbon backbones of most radical polymers are aqueous insensitive. Polyethers like polyethylene oxide (PEO or PEG) and acrylamide are two potential resins. It is possible to stack hydrophilic moieties inside of an otherwise unsaturated radical polymerized polymer like Polyethylene glycol diacrylate (PEG‐DA), where the PEG elements are highly swellable. The formulation challenge is to resolve how many PEG links are needed in a copolymer to achieve sufficiently rapid swelling with an appropriate total swelling response. Acrylamide resins are also very water sensitive, though with longer equilibration times on the order of a week. Natural acids such as citric or alginic acid that can polymerize are also potential candidates. It is much more common to find swelling‐responsive backbone elements in copolymers of one form or another. Polyurethanes (used in Aquavene) remain excellent candidate polymers for expandable IV designs and could be reengineered with new variants on the precursors. The type of the original catalysts for the polyurethane formation for Aquavene was unknown, and since then, new forms of organic catalysts have displaced transition metal catalysts based on platinum and tin so perhaps new polyurethanes are also more biocompatible. There is also substantially more open molecular design with condensation polymers such as polyurethanes. Other segments and chain extenders can be co‐formulated allowing one to tune the swelling response, a feature that makes polyurethanes very attractive. One design could be co‐formulating polyurethanes directly with other components from Table 1, like PEG, but that was the original basis for the Aquavene. Regardless, some revisiting of similar co‐formulations is warranted. New attempts at molecular alchemy could result in new co‐polymeric resins and conceptually new polymers, even if the reaction chemistry remains the same.

It could well be the case that the designed swelling response is somewhat incompatible with sufficient strength and stiffness in a single product. Aquavene was designed as a co‐extruded product and this helped maintain catheter structural integrity, even in the swollen state. Similar strategies to maintain strength could be achieved by extruding around axial fibers. Increased structural strength is crucial to incorporate structural support as highly swollen resins are soft and more likely to buckle when saturated. It is crucial to focus on evaluating candidate materials for structural compliance. Overall, the desirable response characteristics for an ideal expandable IV catheter entails having a high swelling internal diameter rehydration change, a stiffness sufficient for insertion, and to be safe enough to the use environment.

## 5 REDEVELOPING THE EXPANDING IV

Placing and troubleshooting IV catheters is commonplace for medical providers. These catheters were successfully created and found a clinical following, however, a series of acute hypersensitivity‐like adverse reactions caused their early demise. IV catheters are critical in medical practice and despite advancements in placement technologies, IV placement can be difficult. A safer form of expanding IV catheter is yet another tool medical providers could leverage. Given the uncertainty surrounding the cause of the hypersensitivity‐like reactions associated with Aquavene, redeveloping an expanding IV catheter system will be a challenge based on existing biases. .

It is unfortunate that the comprehensive post‐mortem analysis could not determine a cause for the adverse reactions attributed with Aquavene‐containing catheters. Catheter materials, manufacturing practices, and placement protocols could each have led to the observed hypersensitivity reactions. Investigating alternative materials that have appropriate swelling responses is a good step in redevelopment as other resins may not trigger the same hypersensitivity responses. PEG‐DA and acrylamide are potential candidates for replacing the polyurethane‐based Aquavene. If too soft or too swellable, other schemes to reinforce the tubes seems achievable. The softening effects of the Aquavene catheters allowed size increase but questionable rigidity and may have played a role in the higher than normal rate of vein phlebitis seen in patients using these products. At the resin level, varying composition can result in tube‐like shapes that can be produced with variable swelling and mechanical response. Thus, it would be ideal to resolve any link between sensitivity and response before a comprehensive expandable catheter redesign. This would reduce the chance that labile species such as machine oils are the cause of the adverse cellular responses. If lubricants commonly used for polymer extrusion were the overall cause of adverse reaction, then Aquavene materials may actually be safe for use, and a redesign may not be necessary. Without knowing, all future designs risk suffering the same fate as Aquavene.

Important steps forward in any new expandable catheter will include selecting new appropriate polymer compositions for an alternative swelling species by evaluating swelling responses and stability, dynamic mechanical properties with exposure, blood contact compatibility, and identifying possible contaminants in fabrication, assembly, and use. A successful redesign will require investment and effort to how a new design will not result in a similar failure and yield an impactful clinical product. The regulatory bar for a new expanding catheter could be even higher given the failure of the Aquavene catheters that found their way to commercial use.

## CONFLICT OF INTEREST

The authors declare no conflicts of interest.

## AUTHOR CONTRIBUTIONS

Conceptualization: Ariella Shikanov, Leonard A. Mermel, Brian Love, Michael L. Burns

Funding acquisition: Ariella Shikanov, Brian Love, Michael L. Burns

Investigation: Rigoberto Vazquez, Rishabh Tennankore, Ariella Shikanov, Leonard A. Mermel, Brian Love, Michael L. Burns

Resources: Ariella Shikanov, Brian Love

Supervision: Ariella Shikanov, Brian Love, Michael L. Burns

Writing ‐ original draft preparation: Rigoberto Vazquez, Brian Love, Michael L. Burns

Writing ‐ review and editing: Rigoberto Vazquez, Rishabh Tennankore, Ariella Shikanov, Leonard A. Mermel, Brian Love, Michael L. Burns

All credited authors have read and approved the final version of the manuscript.

The corresponding author confirms to have had full access to all of the data in this study and takes complete responsibility to the integrity of the data and the accuracy of the data analysis.

## TRANSPARENCY STATEMENT

This manuscript is an honest, accurate, and transparent account of the study being reported. No important aspects of the study have been omitted. Any discrepancies from the study as planned (and, if relevant, registered) have been explained.

## Data Availability

Data sharing is not applicable to this article as no new data were created or analyzed in this study.
